# Esophagomediastinal Fistula Closed Endoscopically in a Young Patient With Tuberculosis and Human Immunodeficiency Virus

**DOI:** 10.7759/cureus.34813

**Published:** 2023-02-09

**Authors:** Vincent Wong, Ahmed Ahmed, Anjella Manoharan, Weizheng Wang

**Affiliations:** 1 Internal Medicine-Pediatrics, Rutgers University New Jersey Medical School, Newark, USA; 2 Gastroenterology and Hepatology, Rutgers University New Jersey Medical School, Newark, USA

**Keywords:** mediastinal lymph node, immunocompromised patient, tb – tuberculosis, esophageal fistulas, upper endoscopy

## Abstract

Tuberculosis is a primary lung disease that can spread to the lymph nodes, vertebrae, and gastrointestinal tract. The esophagus can be affected by mediastinal lymphadenitis, mostly in immunocompromised patients, leading to the formation of esophagomediastinal fistulas. They can cause dysphagia, pleuritic chest pain, and choking coughs from recurrent aspiration. The treatment is surgery but endoscopic interventions using over-the-scope endoclips, stents, medical adhesives, and sutures are successful alternatives. We present a case of an esophagomediastinal fistula in a patient with tuberculosis and human immunodeficiency virus that was successfully treated with through-the-scope endoclips.

## Introduction

Tuberculosis (TB) is a primary lung disease affecting 10 million people worldwide [1]. Extrapulmonary manifestations affect the lymph nodes, vertebrae, and gastrointestinal tract [2,3]. The esophagus is the least commonly involved and can manifest as fistulas with the trachea, bronchus, and mediastinum [2,4]. These are very rare complications but are seen in immunocompromised patients, such as those with human immunodeficiency virus (HIV) [3,5]. We present a case of a patient with TB and HIV who developed an esophagomediastinal fistula which was treated with through-the-scope endoclips.

## Case presentation

A 29-year-old male with recently diagnosed HIV and TB presented with worsening dysphagia for the past month. His symptoms started suddenly with difficulty swallowing solids and thin liquids as if the food was getting stuck in his throat. He had to forcefully swallow his meals but that led to pleuritic chest pain, violent coughing fits, and post-tussive emesis. He denied any recent fevers, chills, hemoptysis, palpitations, abdominal pain, hematuria, melena, or hematochezia.

Two months prior, the patient was found to have HIV with CD4 of 185 cells/µL at an outside hospital. CT scan also showed mediastinal lymphadenopathy which was biopsied percutaneously and diagnosed as TB. He was started on antiretroviral and anti-tuberculosis therapies at the time.

**Figure 1:**
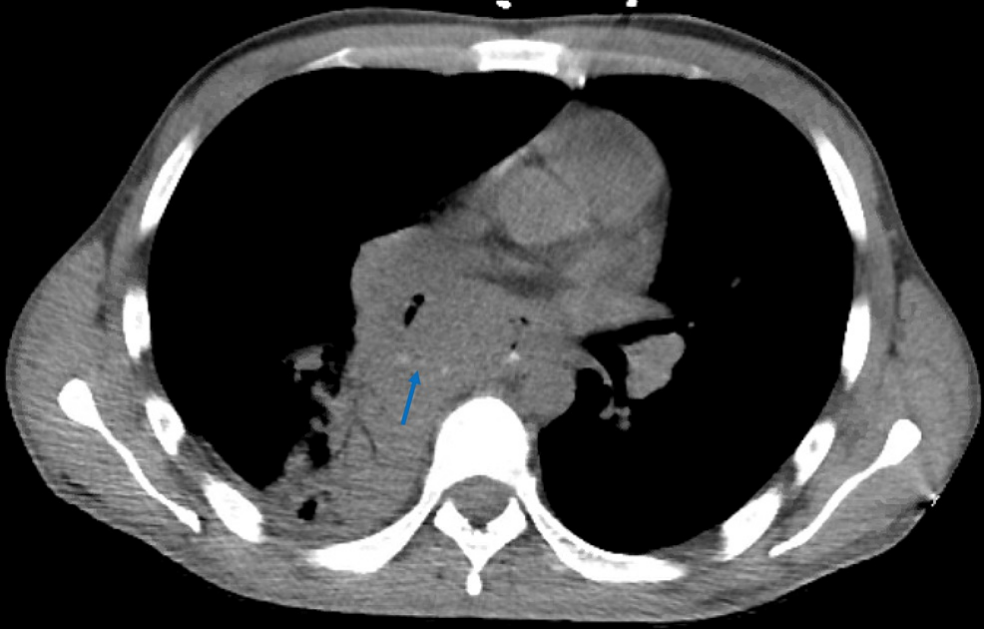
CT scan showing contrast extravasation (blue arrow) into mediastinal mass (1.42x).

On presentation, his vitals were stable, and his basic labs were within normal limits. On physical exam, he was cachectic and in no acute distress with a healed scar below the sixth rib at the biopsy site. His respiratory exam was significant for diminished breath sounds in the right lower lung field with decreased effort due to chest pain upon inspiration. CT scan of the chest showed trace extra-luminal contrast to the right of the esophagus with a small focus of gas in a walled-off non-rim enhancing area of soft tissue within the mediastinum, concerning for an esophagomediastinal fistula (Figure 1).

**Figure 2:**
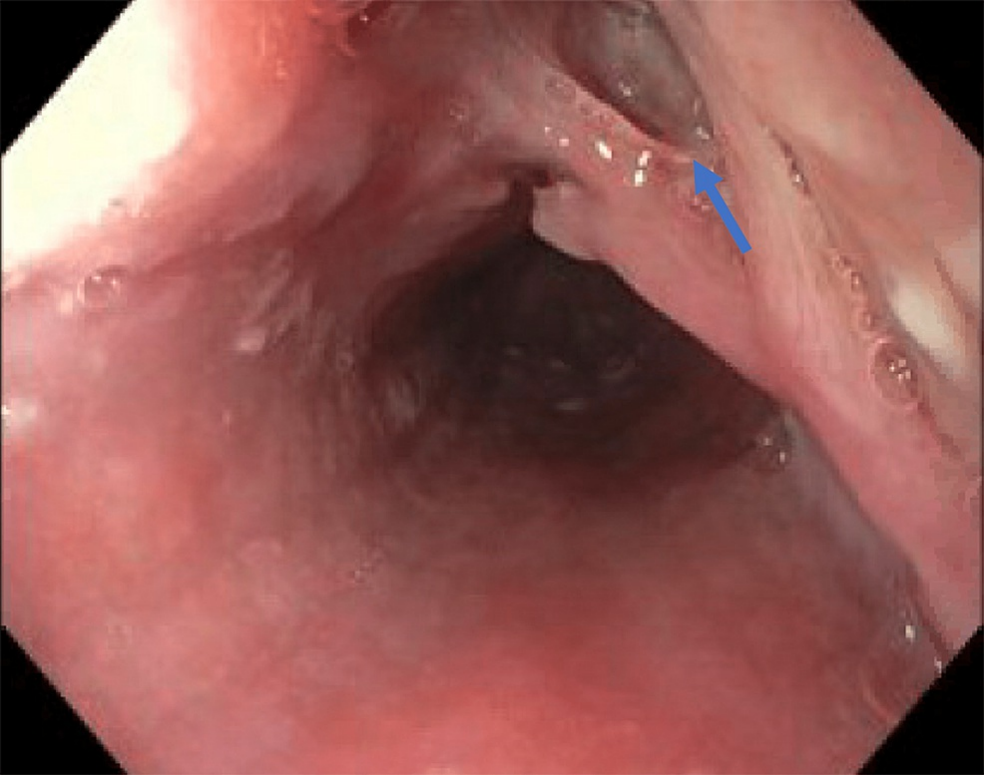
Fistula seen (blue arrow) in the middle third of the esophagus on EGD.

**Figure 3:**
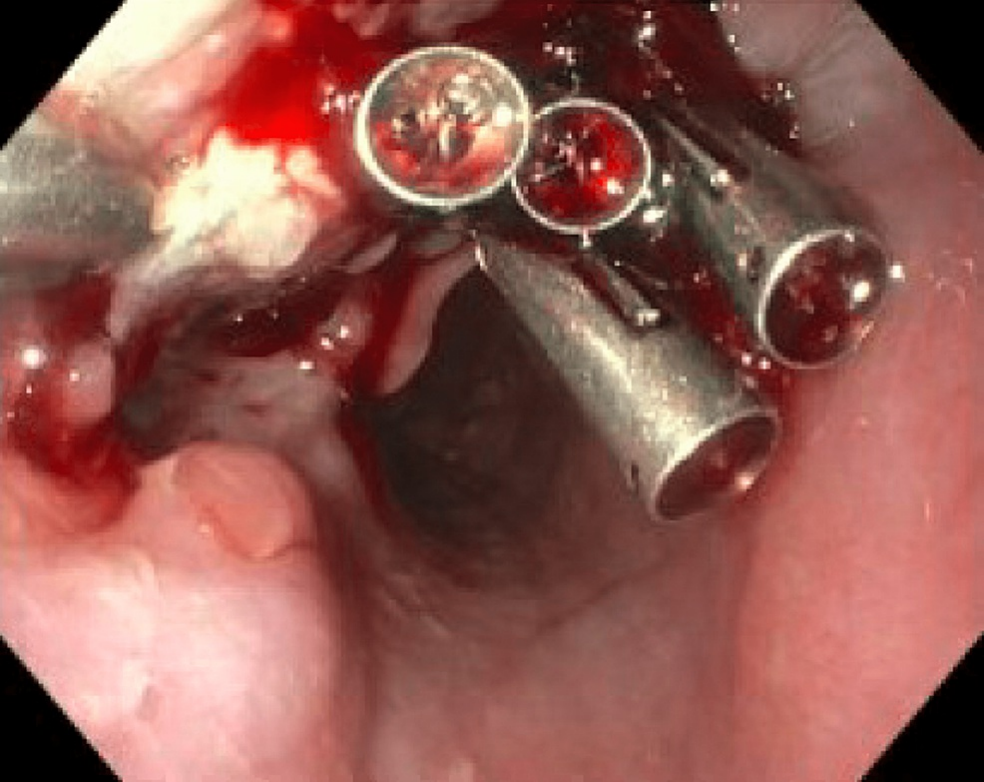
Fistula in the middle third of the esophagus with clips closing the defect on EGD.

**Figure 4:**
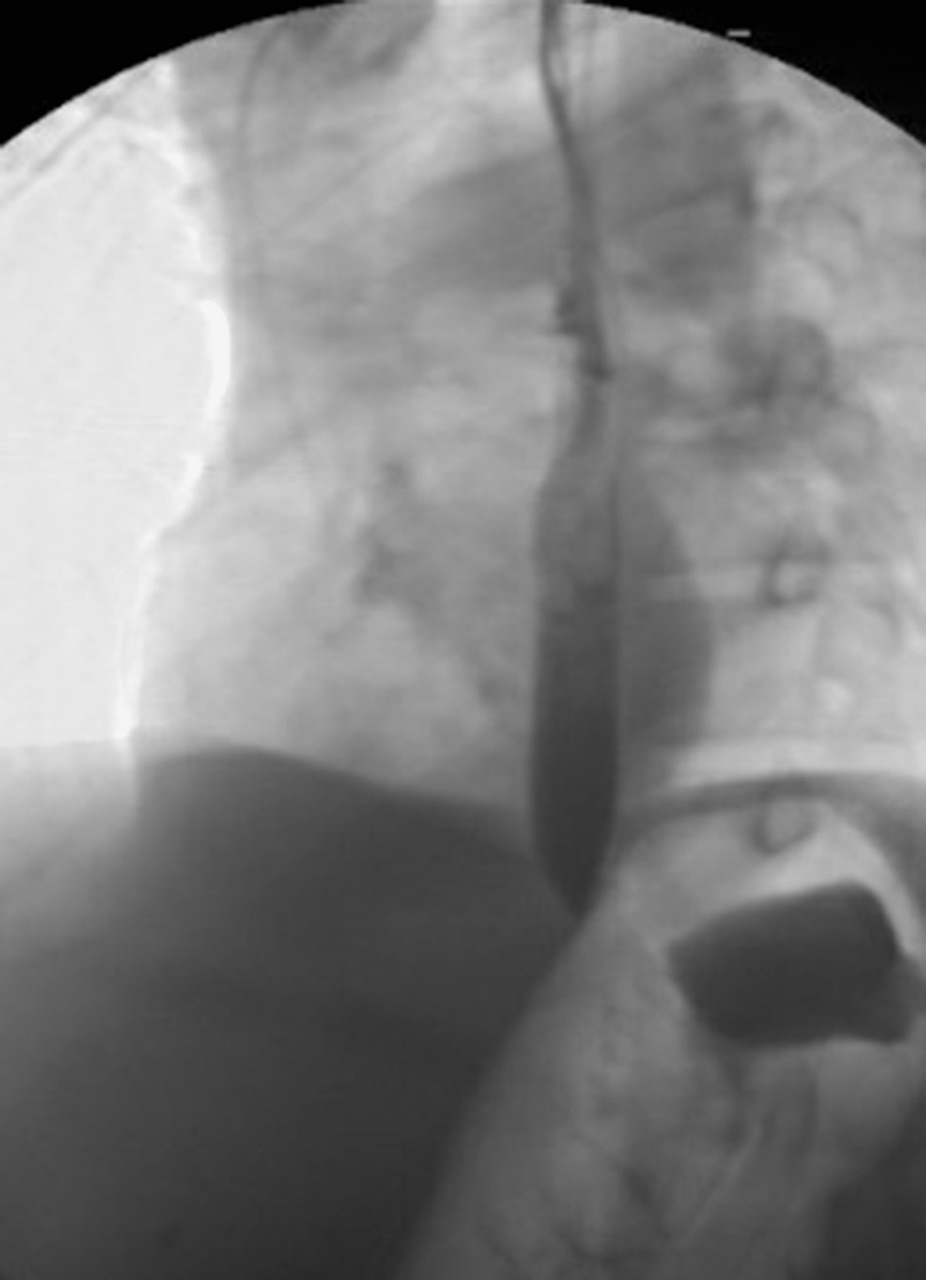
Esophagram showing no signs of contrast extravasation after endoscopic clips were applied to close the fistula.

Esophagogastroduodenoscopy (EGD) was done which revealed esophagitis without bleeding and a fistula in the middle third of the esophagus (Figure 2). Six through-the-scope endoclips were placed to close the defect (Figure 3). A follow-up gastrograffin study one day post-procedure did not show any contrast extravasation (Figure 4).

The patient had a percutaneous jejunostomy tube placed to optimize nutrition and fistula healing. He was discharged on antiretroviral therapy (Efavirenz, Emtricitabine, Tenofovir), anti-tuberculosis medications (Rifampin, Isoniazid, Pyrazinamide, Ethambutol), Pyridoxine, Bactrim for prophylaxis, and Pantoprazole for reflux esophagitis. At his outpatient follow-up two months later, the patient's symptoms resolved and repeat EGD showed that the fistula had completely closed.

## Discussion

Esophagomediastinal fistulas are rare in patients with tuberculosis, reported in approximately 20 cases over the last 30 years [2-12]. They form as a consequence of lymphadenitis, which occurs in about 17.6% of patients with tuberculosis and up to 35% in those with concomitant HIV [3-5]. Tracts develop from ruptured caseating necrotic lymph nodes that compress and erode into adjacent organs, either spontaneously or from iatrogenic procedures [2-4,13,14]. Fistulas can connect the esophagus with the mediastinum and lead to repeated episodes of aspiration [3,5,13]. Patients initially present with a choking cough from eating or drinking [3,5,13]. The diagnosis can be made using an esophagram, high-resolution CT, or EGD [3,5,13]. The mainstay of treatment is to close the defect, control the infection, and improve nutritional status [3,5,13]. These types of fistulas are usually surgically ligated or excised [3,15]. However, they can be closed endoscopically using clips, metal stents, medical adhesives, and stitches [6,7,13-20].

In the literature, over-the-scope (OTS) endoclips have been used to close fistulas in patients that have larger defects of up to 3 centimeters (cms), with a success rate of 89% [16,17,20]. However, they require additional setup time to load onto the endoscope, and removal of the clips is often difficult [17]. Metallic or plastic self-expanding stents can be used with up to 83% success rate but carry risks for stent migration (10%-30%) and luminal perforation (3%) [17]. Fibrin glue is another modality for fistula closure but there are reports of the glue clotting inside the injection catheter, and being aspirated into the airway [7,16,17]. Endoscopic suturing can close defects larger than 2 cm and has a greater than 95% success rate but is technically challenging to perform and can reopen in 65% of patients [16,17]. On the contrary, through-the-scope (TTS) clips have mainly been used for hemostasis and perforations less than 2 cm provided that the surrounding mucosa is healthy [8,16]. With recent technology, they are easy to load, rotatable, reusable, and can be maneuvered into difficult-to-reach areas [16,17]. The use of TTS endoclips described in this case demonstrates their effectiveness in closing esophagomediatinal fistulas [6].

Our patient developed an esophagomediastinal fistula shortly after his diagnosis of tuberculosis. Based on the timing of his symptoms, the etiology is likely multifactorial. TB could have caused lymphadenitis, which was further exacerbated by the biopsy, and subsequent remodeling in the setting of a compromised immune system likely led to fistula formation. Through-the-scope endoclips were used in this case because it was easy to load into the endoscope and rapidly deployable. A jejunostomy tube was placed to maximize nutrition in our cachectic patient but also to prevent worsening reflux that can affect the healing process. The patient was compliant with his antiretroviral and antituberculosis medications. His fistula closed after two months making it one of the first cases of a successfully treated TB-related esophagomediastinal fistula with through-the-scope endoclips [6].

## Conclusions

For immunocompromised patients with tuberculosis who present with dysphagia, a thorough history and physical exam should be done to determine the presence of an esophagomediastinal fistula. They can be treated either surgically or endoscopically, but this case suggests that through-the-scope endoclips can be another alternative. They are minimally invasive, easy to load, quick to deploy, low risk, low cost, and can lead to complete fistula closure.
